# Predictors of time-to-recovery from severe acute malnutrition treated in an outpatient treatment program in health posts of Arba Minch Zuria Woreda, Gamo zone, Southern Ethiopia: A retrospective cohort study

**DOI:** 10.1371/journal.pone.0234793

**Published:** 2020-06-30

**Authors:** Kidane Gebremedhin, Gistane Ayele, Negussie Boti, Eshetu Andarge, Teshale Fikadu

**Affiliations:** 1 Mirab Abaya District Health Office, Gamo Zone Health Department, Gamo Zone, Southern Ethiopia; 2 School of Public Health, College of Medicine and Health Sciences, Arba Minch University, Arba Minch, Southern Ethiopia; University of Minnesota, UNITED STATES

## Abstract

**Background:**

Access to outpatient therapeutic feeding programs for all children who had uncomplicated severe acute malnutrition (SAM) in need is a global health priority. In Ethiopia SAM is treated in hospitals, health centers and health posts. Health extension workers (HEWs) manage SAM that is uncomplicated at the health posts through the outpatient therapeutic feeding programs (OTP). Identifying predictors that predict time-to-recovery of children on OTP is thus vital to optimizing therapeutic success. However, the factors affecting children’s’ recovery time at this peripheral health institutions were not well documented. Therefore, this study aimed to identify predictors of time-to-recovery from SAM among children treated at an OTP in health posts of Arba Minch Zuria woreda, Gamo Zone, Southern Ethiopia.

**Methods:**

A retrospective cohort study was conducted on 402 children enrolled in an OTP in the health posts of Arba Minch Zuria woreda based on data abstracted from their medical records. The study children were selected using systematic random sampling method using a list of their medical record numbers. Both descriptive and analytic analyses were performed. Median time of recovery was estimated by using the Kaplan-Meier survival curve. Furthermore, bivariate and multivariable Cox proportional hazard regression analyses were used to identify factors significantly associated with outcome variable.

**Result:**

The median time-to-recovery from severe acute malnutrition among children was 49 days (Interquartile range [IQR]: 42–56). Among the participants, 70.40% with 95% CI: (74.2–85.0%) recovered from severe acute malnutrition. The Cox-proportional hazard analysis showed that children’s age at admission (Adjusted hazards ratio [AHR] = 3.15; 95% confidence interval [CI]: 1.85, 5.03), diagnosis with edema (AHR = 1.75, 95%CI: (1.27, 2.43), co-morbidity of diarrhea (AHR = 0.22, 95% CI(0.13, 0.39), and anemia (AHR = 0.64, 95% CI:(0.42, 0.98) were found to be predictors of time to recovery from SAM.

**Conclusions:**

The median time-to-recovery at the health posts in this study was in the accepted time period for the maximum Ethiopian standard protocol set for the management of SAM. However, the nutritional recovery rate was lower than the minimum acceptable threshold for the Sphere International Standards. Therefore, early screening of co-morbidity like diarrhea, anemia and edemaand timely intervention would increase the chance of recovery of children.

## Background

Malnutrition is a condition that results from deficiencies, excess or imbalance in a person’s energy intake and/or nutrients [1]. Acute malnutrition is the most severe form of malnutrition potentially affecting all categories of the population especially children under five years of age [2–4]. Severe acute malnutrition is one of the forms of acute malnutrition which is defined as below 3 standard deviations (SD) of the WHO growth standards or weight for height(W/H) <70% of the median National Center for Health Statistics standard and the presence of bilateral pitting edema or Mid-Upper Arm Circumference(MUAC) <11cm [5].

Today, SAM is a global public health problem that majorly affects the survival of children under five years of age [3]. Globally, an estimated 555 million children were under five years of age and of these 52 million were suffering from acute malnutrition. More than 90% of those with acute malnutrition live in the developing countries [3]. According to the 2019 Ethiopian mini Demographic and Health Survey (EDHS) report, 37%, 21% and 7% of children under 5 years of age were stunted, underweight and wasted in Ethiopia respectively. In Southern Ethiopia, 36.3%, 19.7% and 6.3% were stunted, underweight and wasted [6].

SAM is one of the main risk factors for morbidity and mortality among children [3,7]. It has also significantly contributed for impaired intellectual development of children, increased the risk for disease and it is one of the main reasons for children’s hospital admission [8]. The risk of death is 9 times higher for children with SAM than that of children without SAM [9]. Each year about 3 million children with SAM will die due to its effect in decreasing their immunity against common infections as it increases the severity and frequency of the infections and delays the recovery from the infections [3].

Until recently, the management of SAM has been delivered solely at hospitals with limited coverage and accessibility to the cases [10]. However, only 10% of the affected children got treatment from hospital [11] More than 85% of children with SAM without medical complications were managed at home [12]. In response to this, a new management approach –community management of acute malnutrition (CMAM) programme–was started to make services accessible and available to the community in most developing countries [10,13,14].

In Ethiopia, the CMAM Program was first piloted in Southern nations, nationalities and peoples region (SNNPR) in 2004 [13]. However, the service was decentralized to the health posts level starting from 2008 [15].

According to the new guideline, SAM is managed either in an outpatient or inpatient basis [3,16,17]. The OTPs are part of the routine health care services to treat malnutrition that has been provided at health centers and health posts. The health care services include diagnoses of children with SAM within health facilities and at community level by health extension workers (HEWs), provide ready-to-use therapeutic foods (RUTF), usually Plumpy’Nut that has been given every week to be eaten at home and a course of routine medications including amoxicillin, folic acid, vitamin-A, measles vaccine and de-worming. Only children who don’t have medical complications and have passed the appetite test with Plumpy’Nut are eligible to the OTP [7].

Plumpy'Nut is a ready-to-use therapeutic spread produced by Nutriset and presented in individual sachets. It is a paste of groundnut composed of vegetable fat, peanut butter, skimmed milk powder, lactoserum, maltodextrin, sugar, mineral and vitamin complex [7]. For appetite test, Plumpy’Nut was offered to each child. If a child had a body weight of <4kg, then she/he was offered an amount of 1/8-1/4 sachet, likewise, 1/4-1/2 sachet if she/he had body weight of 4 to 10 kg, ½-3/4 sachet for 11 to 15 kg and ¾-1 sachet for >15 kg. The child was considered to have passed the appetite test if she/he consumed the required amount according to his/her body weight within 30 minutes. If the child to the minimum requires ready to use therapeutic food (RUTF)he/she will be referred to in-patient care [18,19].

Contrary to these efforts, studies conducted in different parts of the country revealed that the recovery rates and median survival times of children under five years of age with SAM ranged from 51.9% to 79.8% and 16 days to 71 days, respectively [12,20–22]. Evidences also showed that in Ethiopia the recovery rate among children attending the OTPs was still low as compared to the acceptable minimum standard, and this has a negative impact on children’s health and survival [8,23,24].

The predictors of time to recovery from SAM identified by different previous studies were having anemia at admission, maternal illiteracy, sharing of RUTF, co-morbidity with diarrhea and age of the child [20,22,25]. However, the majority of previous studies were conducted in hospitals and health centers. To the best of our knowledge, there is a dearth of evidence on the predictors of time-to-recovery from SAM at the health posts (the utmost peripheral health institutions affiliated by community HEWs) level. Since predictors vary from context to context and the predictors of recovery in such peripheral institutions would imply an insight for interventions, the findings of this study would assist in health planning and policy making for the better outcome of the CMAM. Therefore, this study aimed to identify predictors of time to recovery from SAM among children 6–59 months of age using the OTP at rural health posts of Arba Minch Zuria woreda, Gamo zone, Southern Ethiopia.

## Methods

### Study setting and design

An institution-based retrospective cohort study was conducted from January 1, 2016 to December 31, 2018 in the health posts of Arba Minch Zuria woreda, Gamo zone, Southern Ethiopia. According to Gamo zone health office report; there were 14 rural woredas and 4 city administrations in the zone having three different climatic zones: high-land, mid-land and low-land. [*Woredas are the fourth-level administrative divisions of the Ethiopian government structure under zones in the regional governments of the country which are assumed to be comparable to districts in the other parts of the world]*. Arba Minch Zuria woreda is one of the rural woredas in Gamo zone. As its name implies, it is the woreda surrounding Arba Minch town, the main town of Gamo zone. The woreda has a total of 31 kebeles (*Kebele is the fifth- level administrative unit under woredas in the Ethiopian government structure which is the smallest administrative unit in the structure)*. The estimated number of children 6–59 months in the year 2016/17 in Arba Minch Zuria woreda was 15,484.

The major source of livelihood in the woreda is crop production, livestock rearing, petty trade, and unskilled labor. The major cash crops grown are maize, barely, ‘*enset*’, coffee, ‘*khat*’, wheat, and bean [26]. During the data extraction from medical records, data of children whose date of admission, date of discharge and/or type of diagnosis were not recorded and those who were transferred in from other health facility were excluded.

### Sample size determination

The sample size for this study was calculated based on two population proportion formula using Epi-Info-7.2 Stat Calc software. The following assumptions were considered: 95% confidence interval for a two-sided test, 80% power with a minimum detectable alternative of ± 5%, ratio of unexposed to exposed of 1:1, the proportion of children with diarrhea who were recovered (exposed group) = 47.4% and the proportion of children without diarrhea who were recovered (non-exposed group) = 65.9% [21]. Accordingly, the calculated sample size was 246 participants. After consideration of a design effect of 1.5 to compensate for cluster sampling and addition of 10% of the sample for missing and incomplete data, the total sample size required for this study was 402 participants.

### Procedures of enrollment to the OTPs

First, children are identified by health extension workers using MUAC and edema criteria according to the national protocol. The presence of any complication is early screened for treatment by health extension workers. Then appetite test is performed. Health extension workers discuss with caregivers and decide upon the appropriate treatment options. Children having major medical complications are referred to the nearby health center for admission while those having good appetite and no major medical complications are enrolled in the OTP at the health posts. In the health posts, children are followed for maximum of eight weeks visiting the facilities per week and receive routine medications and monitored for the progress of their condition.

### Sampling procedures

**Fig 1:**
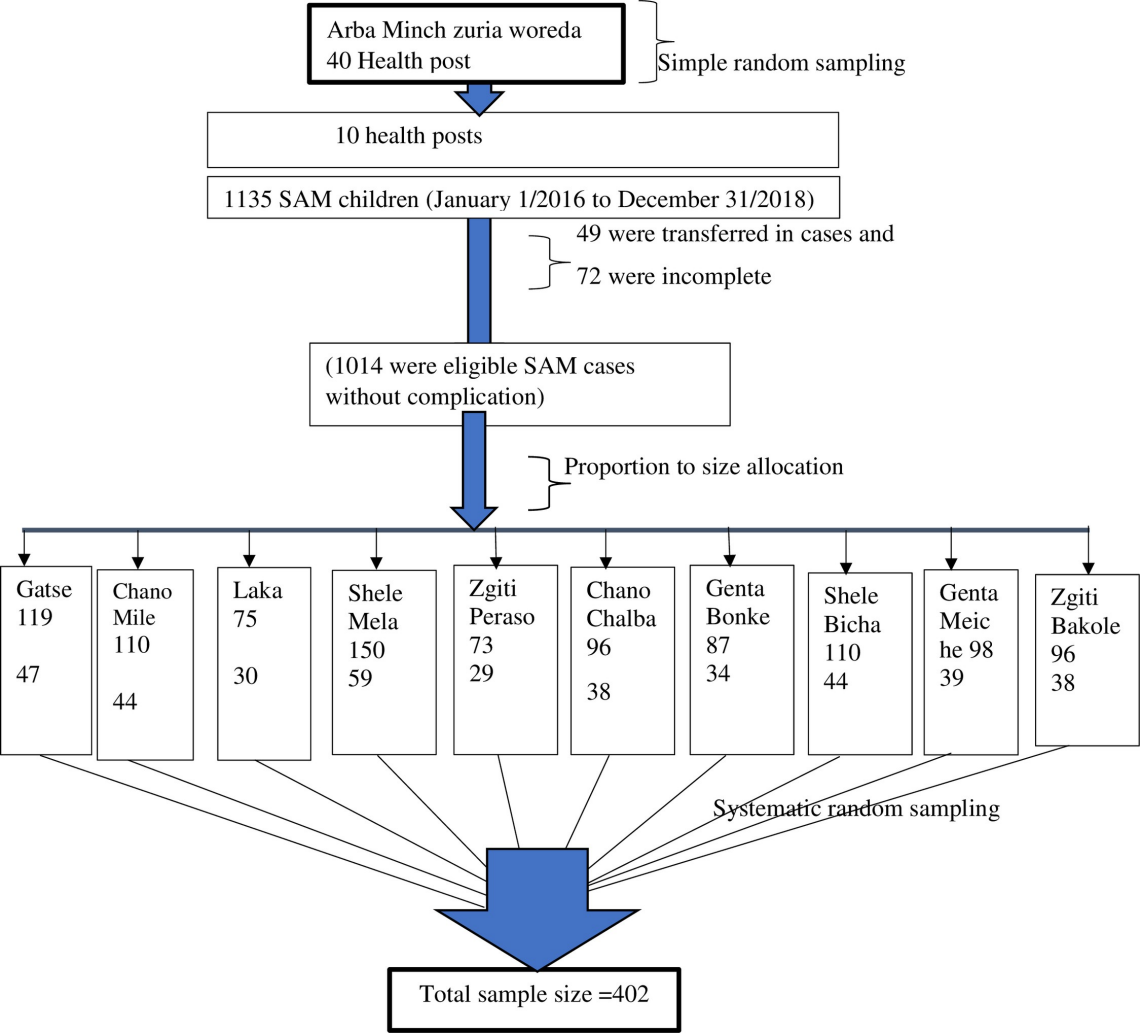
Schematic presentation of the sampling procedure.

Arba Minch Zuria woreda has a total of 40 health posts. Each health post had an average of 5,000 catchment populations. Among the 40 health posts, 10 health posts were selected by a simple random sampling technique. The sampling frame was prepared for each selected health post through identifying the eligible children with SAM from the registration logbook. Then the total sample size was allocated to the selected health posts in proportion to their size of children with SAM. Finally, a systematic random sampling was employed to select the participants of the study for each health post from the list of children’s medical registry number obtained from the respective health posts **(Fig 1).**

### Variables of the study

The Outcome variable of the study was time-to-recovery from SAM (i.e. the event of interest is recovery and that the response variable was the rate of recovery). The independent variables considered were age of children, sex of children, distance from the OTP sites, type of malnutrition (marasmus or kwashiorkor) and clinical symptoms (diarrhea, anemia, cough, and fever).

### Definitions and measurement

**‘**Enset’, with the scientific name Ensete ventricosum, Ethiopian banana, Abyssinian banana, and false banana, is an herbaceous species of flowering plant in the banana family Musaceae[27].

Khat or qat is a flowering plant native to the Horn of Africa and the Arabian Peninsula. Khat contains the alkaloid cathinone, a stimulant, which is said to cause excitement, loss of appetite, and euphoria [28].

Recovery was defined based on national protocol and global Sphere standards criteria used to diagnose SAM upon enrollment. If children were admitted based on edema, recovery was the resolution of edema at two consecutive weeks while if the children were admitted based on low MUAC, MUAC > 110 mm at two consecutive weeks and/or achieving target weight gain within the maximum stay of 8 weeks in the OTP program. However, if the children failed to achieve the aforementioned recovery criteria within the maximum eight weeks treatment and missed appointments for two consecutive weeks while being confirmed that they were alive were considered as censored [12,21,25].

Time-to- recovery was determined by calculating the differences in the number of days it took from date of initiation of treatment until a child was recovered from SAM.

### Data collection procedure

The data extraction tool was adapted from Ethiopian protocol for the management of SAM and the sphere standard for the management of SAM [7,22,29]. Data for this study were extracted from OTP cards by eight experienced nurses and supervised by three public health officers after two days of intensive training.

### Data quality control

The study checklist was pre-tested on 5% of sample size (21 samples) in one health post outside the selected ones to ensure clarity on wordings, logical sequence and skip patterns of the questions. Based on the pretest, the time needed to complete the checklist and the total numbers of days needed for data collection were estimated. An intensive training was provided to data collectors and supervisors that included briefing on the data collection process of the study, discussing the contents of the checklist. The overall activity of data collection was supervised and coordinated by the supervisors. The collected data were reviewed and checked for completeness and consistency and feedback was provided on daily basis for on-site corrections. During data entry and analysis, data were coded and checked using Epi-data and preliminary cleaning was done using STATA for missing values and inconsistencies through checking frequencies.

### Data processing and analysis

Data were coded, checked and entered into Epi Data version 4.4 software and exported to STATA version 13 for cleaning and analysis. Descriptive analysis was carried out and presented using frequencies, percentages and measures of central tendency and dispersion. Kaplan-Meier survival curve was used to estimate the median duration of recovery. A log-rank test was used to compare recovery curves for different categories of predictors. Bi-variable and multivariable Cox-proportional hazard models were used to identify predictors of time-to-recovery. Those variables that had a p-value less than 0.25 in the bi-variable Cox-proportional hazard model were considered as candidates for the multivariable Cox-proportional hazard model. Both crude and AHR with the respective 95% confidence intervals (CI) were reported and interpreted to show the degree of association between time-to-recovery and the explanatory variables. The proportional hazard assumption of the model was assessed based on Schoenfeld residuals test. Multi-collinearity between explanatory variables was checked using variance inflation factor (VIF>10).

### Ethics consideration

Ethics clearance was obtained from the institutional review board of College of Medicine and Health Sciences, Arba Minch University. Permission to conduct the study was obtained from Gamo zone health department and Arba Minch Zuria woreda health office as well as from HEWs of the respective health posts selected for the study. Confidentiality to information was maintained through not using any personal identifiers on the checklists for data collection and the recorded data was kept away from access to a third person but for the team of investigators.

## Results

### Socio-demographic and admission characteristics of respondents

Out of the 402 child records reviewed, 226 (56.22%) were male and 176 (43.78%) were females. The records of the children also showed that 132 (32.84%) of them belong to ≤ 24 months of age, 270 (67.16%) of them belong to >24 months and they were in the age range of 7-57 months. Sixty-eight (16.92%) of them were breastfeeding whereas 334 (83.08%) were not breast feeding. More than half (59.70%) of the children had edema and 162 (40.30%) were diagnosed as having had a marasmus (Table 1).

**Table 1:** Socio-demographic and admission characteristics of children admitted to OTP from SAM, Arba Minch Zuria woreda, Southern Ethiopia, 2019 (n = 402).

Child characteristics	Category	Frequency	Percent
Sex	Male	226	56.22
	Female	176	43.78
Age	≤ 24 months	132	32.84
	>24 months	270	67.16
Breast-feeding status	Yes	68	16.92
	No	334	83.08
Admission status	New admission	324	80.60
	Re-admission	78	19.40
Distance (Time of travel in minutes)	Less than 30 min	238	59.20
	More than 30 min	164	40.80

### Medical co-morbidity and routine medication provision of children with SAM

Of the total 402 children, 240 (59.70%), 162 (40.30%), 350 (87.06%), 136 (33.83%), 71 (17.66%) and 45 (11.19%) of children had edema, marasmus, fever, diarrhea, pneumonia and anemia respectively. The overall median weight at admission was 7 kg (IQR: 6.1 to 9 kg). The median weight was 7.5 kg (IQR: 6.15–10 kg), and 7 kg (IQR: 6.1–9 kg) for children with marasmus and edema respectively. Three of the most commonly given medications were measles vaccine, vitamin A, and antibiotics for 94.07%, 85.5%, and 81.09% of children respectively (Table 2).

**Table 2:** Medical co-morbidity and routine medication provision of children with SAM on admission under OTP in health post of Arba Minch Zuria woreda, Southern Ethiopia, 2019.

Variables	Categories	Frequency	Percent
**Admission criteria**	Edema	240	59.70
	Marasmus	162	40.60
**Fever**	Yes	350	87.06
	No	52	12.94
**Malaria**	Yes	58	14.43
	No	344	85.57
**Cough**	Yes	46	11.44
	No	356	88.56
**Diarrhea**	Yes	136	33.83
	No	266	66.17
**Vomiting**	Yes	28	6.97
	No	374	93.28
**Pneumonia**	Yes	71	17.66
	No	331	82.34
**Anemia**	Yes	45	11.19
	No	357	88.81
**Dermatosis**	Yes	33	8.21
	No	369	91.79
**Failed appetite test**	Yes	57	14.18
	No	345	85.82
**Provided with vitamin A (n = 367)**	Yes	313	85.29
	No	54	14.71
**Provided with folic acid (n = 402)**	Yes	34	8.46
	No	368	91.54
**Provided with deworming (n = 334)**	Yes	244	70.05
	No	90	26.95
**Provided with anti-malaria drugs(n = 402)**	Yes	58	14.43
	No	344	85.57
**Provided with antibiotics (n = 402)**	Yes	326	81.09
	No	76	18.91
**Provided with measles vaccine (n = 337)**	Yes	317	94.07
	No	20	5.93

### Time-to-recovery from SAM

The finding of this study reveals that among 402 children with SAM; 283 (70.40%) were recovered, whereas, 53(13.18%), 36 (8.96%) and 6 (1.49%) children defaulted from the OTP, transferred to inpatient care, and died, respectively. The overall median time-to-recovery was 49 (IQR: 42–56) days.

### Comparison of survival time among categories of covariates

**Fig 2:**
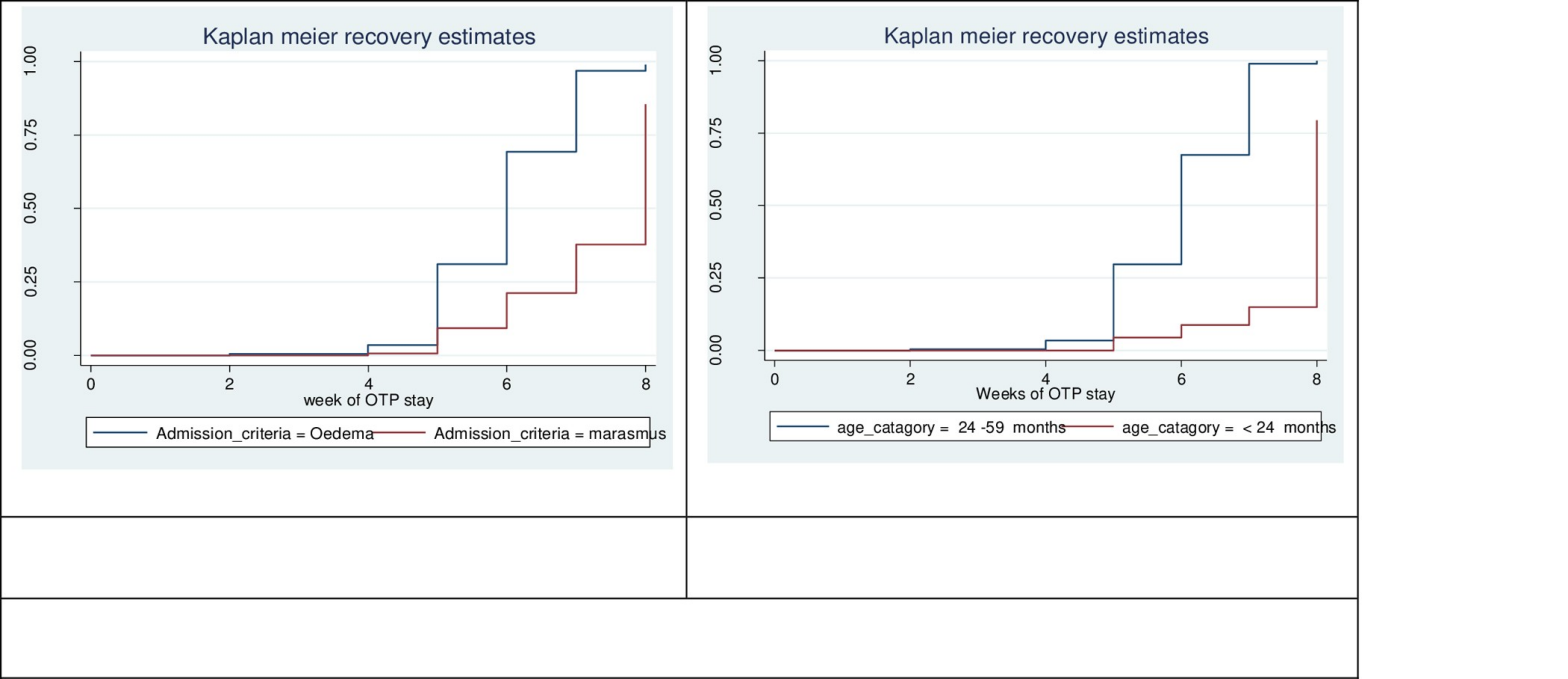
Survival curves for children with SAM by their admission criteria and age at health posts of Arba Minch Zuria woreda, Gamo zone, Southern Ethiopia.

To check for the existence of median survival time differences in between the various categories of variables considered in this study, we used the Kaplan Meier survival curve together with a log-rank test. Accordingly, children with marasmus during admission had significantly longer survival time compared with children admitted with edema (log-rank, p<0.01). Children <24months of age had significantly longer recovery time compared with children ≥24 months of age (log-rank, p<0.05) (Fig 2).

### Predictors of time-to-recovery from severe acute malnutrition

Before fitting the covariate into the model, the proportional hazard assumptions were checked by Schoenfeld residual test and graphically using log minus log plots. None of the variables violated the proportional hazards’ assumptions.

Age of the child at admission, time to travel from home to health post, admission, admission status, admission criteria (Co-morbidity with malaria, diarrhea, anemia, dermatosis)and children’s receipt of folic acid, and measles vaccine were candidates for multi-variable Cox regression model(p-value ≤0.25). To control for possible confounders and identify the independent predictors of time-to-recovery, multivariable cox regression model was fitted using backward step-wise method.

In the final multivariable Cox regression model, children >24 months old during admission were 3.15 times (AHR = 3.15, 95% CI = 1.85–5.03) more likely to recover from SAM as compared with those ≤ 24 months old. Likewise, children diagnosed with edema during admission were nearly 2 times (AHR = 1.75, 95% CI = 1.27, 2.43) more likely to recover from SAM as compared with those diagnosed with marasmus. However, children with diarrhea co-morbidity were 78% (AHR = 0.22, 95% CI = 0.13, 0.39) less likely to recover from SAM as compared with those without diarrhea co-morbidity. Similarly, those children with anemia were 36% (AHR = 0.64, 95% CI = 0.42,0.98) less likely to recover from SAM as compared with those without anemia (Table 3).

**Table 3:** Predictors of time-to-recovery from SAM among children enrolled in the OTP in the health posts of Arba Minch Zuria woreda, Gamo zone, Southern Ethiopia, 2019.

Variables	Category	CHR (95% CI)	AHR (95% CI)
**Age of child**	≤ 24 months	1	
	>24 months	7.35(5.15, 10.51)	3.15(1.85,5.03)^*^
**Distance to health post**	< 30 min	1	
	≥30 min	0.72(0.57,0.92)	0.88(0.68, 1.14)
**Admission status**	New admission	1.21 (0.90, 1.63)	1.07(0.78, 1.46)
	Re-admission	1	
**Admission criteria**	Edema	3.52(2.67,4.65)	1.75(1.27,2.43)*
	Marasmus	1	
**Malaria**	Yes	0.79(0.56, 1.13)	0.86(0.57, 1.31)
	No	1	
**Diarrhea**	Yes	0.09(0.07,0.15)	0.22(0.13,0.39)*
	No		
**Anemia**	Yes	0.67(0.44,1.01)	0.64(0.42,0.98)*
	No	1	1
**Provided with folic acid**	Yes	1	
	No	0.62(0.43, 0.89)	0.89(0.58,1.36)
**Provided with measles-vaccine**	Yes	1.21(0.88,1.15)	1.03(0.87, 1.21)
	No	1	

* Significant association at a p-value of <0.05, 1: set as a reference group, CHR- crude hazard ratio, AHR -adjusted hazard ratio, CI- confidence interval

## Discussion

The primary objective of this study was to identify the predictors of time to recovery from SAM among children aged 6–59 months who were treated in an OTP at health posts of a rural woreda in Southern Ethiopia. The median survival time was 49 days/7 weeks. In the study, age of children, edema at admission, anemia at admission and presence of diarrhea as co-morbidity were identified as the independent predictors of time to recovery from SAM. The median time-to recovery from SAM in this study was longer than the international standard (SPHERE) set for the management of SAM and from findings of a study conducted in Jimma health center and Shebedino, Southern Ethiopia(36 days) [29,30]. But, the median recovery time was in the range of the accepted maximum Ethiopian standard set for the management of SAM which is 8 weeks (56 days) [5]. On the contrary, it was shorter than the study conducted in Dire Dawa, Eastern Ethiopia [12]. The median survival was comparable with findings from studies conducted in Kamba District, South West Ethiopia (50days), North Western Ethiopia (48 days) [20,22,25]. The proportion of the study participants who recovered from SAM in this study was also higher than the findings from the study conducted in Wolaita zone, Southern Ethiopia(64.9%) [21]. On the contrary, this finding is lower than the findings from studies conducted in health post level of Malawi (89%), Shebedino, (78%) and Badawacho(85%) Southern Ethiopia [9,14,20] and the minimum 75% threshold set by the Sphere standard [27]. However, this finding was comparable with the study conducted in Kamba district (68%), Southern Ethiopia and Kenya (73.3%) [14,22]. The disparities in recovery time might be attributable to differences in treatment and caring practices among facilities since most of the previous studies were conducted on data from hospitals, health centers and health posts [22,25] and this study assessed the recovery at health post level. Besides this, the difference might be due to differences in adherence to guidelines to optimal management of children under OTP across regions [4], differences in seasons and setting-specific differences in predictors of time-to-recovery in the different contexts of the studies. The finding might particularly indicate that HEWs at health posts in the periphery of the health care system are able to provide management of SAM at OTPs to the national recommended standards set for median recovery time though efforts are needed to shorten the time-to-recovery to the global standards.

In the present study, the mean weight gain rate during the study period among children managed in the health posts under the OTP program was 4.25 g/kg/day. This finding was below the standard set rate by the international SPHERE which recommends weight gain rate greater than 8 g/kg/day [29]. Also, this finding was comparable with a study conducted among children treated at OTP in Wolaita zone (4.2 g/kg/day) [21]. This finding was slightly lower than the study conducted in Kenya (5.1 g/kg/day), Kamba district (5.76 g/kg/day) and Shebedino district (5.4 g/kg/day) [4,20,22]. The possible reasons for the low weight gain might be associated with late detection of children with SAM that would increase the risk of co-morbidities which in turn would have resulted in the weight gain falling short of the standard. Likewise, this might be associated with lack of routine medications such as Iron and folic acid, antibiotics, and vitamin A [10,31]. On the other hand, the low weight gain might be associated with the common practice of caregivers’ sharing RUTF with other members of the family [4,20]. Even though discrepancies were there among the weight gains observed from the studies in Eastern Africa [20,22,30], all the findings consistently showed a lower achievement in weight gain compared with the international SPHERE standard of 8 g/kg/day.

The finding of this study showed that age of children at admission significantly affected the time- to-recovery among the study participants. The time needed for recovery from SAM among children who are less than 24 months old was longer than those ≥24 months old. This finding corroborates the findings of the study conducted in Kamba, Southern Ethiopia [22]. The observed discrepancy among the groups might be owing to the fact that the first 24 months of a child’s life requires optimal nutrition to meet their nutritional requirements and this might require longer duration of recovery from SAM [11]. Similarly, children under 2 years do not meet the dietary diversity and feeding frequency appropriate to their age as this might prolong the duration of recovery from SAM [11]. Moreover, children who are less than 24 months might be more vulnerable to infections due to immature immunity during the admission that might require, longer time to recovery [3]. This calls for a critical follow-up to these groups of children by health extension workers and other stakeholders.

This study depicted that edema at admission was an important predictor of time to recovery. Children diagnosed with edema at admission were nearly 2 times more likely to recover early than those children with marasmus. This finding is in agreement with findings from the Shebedino and Wolaita studies in Southern Ethiopia, and Tigray, Northern Ethiopia [10,20,21,31]. The difference observed among the groups might be for the fact that children enrolled by the edema criteria resolve their edema easily after OTP which results in shorter time to recovery than those children who were admitted with marasmus. These has an implication to the health extension workers and caregivers to critically follow those children diagnosed with marasmus [23,25].

Likewise, the presence of anemia at admission significantly affected the time to recovery from SAM in this study. Children who had anemia during enrollement were less likely to recover from SAM as compared with those without anemia. The finding is in line with the study conducted in Bahir Dar city, Northwest Ethiopia where children who had not had anemia at admission were more likely to recover compared with those who had anemia [25]. This might be owing to the fact that children who were anemic at admission need longer time to recover till they get iron supplements according to the national protocol for the management of SAM and receive iron supplement can early recover from SAM [21,32]. The finding suggests early screening and due attention from health extension workers and other stakeholders to children with anemia so that a better recovery to the standard can be achieved among children.

The other co-morbidity condition which predicted the time to recovery from SAM was presenting with diarrhea during the admission. Children admitted with diarrhea were 78% less likely to recover from SAM as compared with those children without diarrhea during the admission. This finding was supported by a study conducted in Shebedino, Southern Ethiopia and Tigray, Northern Ethiopia [10,20]. The possible explanation to this association might be children with malnutrition and diarrhea would likely experience loss of the intestinal mucosal barrier that results in systematic immune-suppression [8]; therefore it takes longer time to this children to recover than those children admitted without diarrhea. Besides this, children admitted with diarrhea had a retarded weight due to compromised absorption and increased demand for nutrients [33].

Unlike most of the earlier studies, this study was conducted at health post level in rural kebeles and able to assess independent predictors of time to recovery with a better design that would strengthen the CMAM programme recently under implementation in the country. However, the retrospective nature of the data limited to obtain information on RUTF sharing practices, selling behaviors and food security status commonly reported as predictors of time to recovery.

## Conclusion

The median time-to-recovery was 49 days. Age of children at admission, presence of edema, diarrhea and anemia at admission were independent predictors to time-to-recovery from SAM. Therefore, special focus should be given to young, children with marasmus, diarrhea, and anemia. Besides, there is a need for a prospective study to dig out the causes of longer recovery time by including additional predictors like the provision of the treatment to the malnourished child at home, household income level and mother’s perception towards the disease.

## Supporting information

S1 Data Data extraction tool. (DOCX)

S2 Data The raw data supporting the results of the study. (XLSX)
