# Supplementary material for: Predictors of time-to-recovery from severe acute malnutrition treated in an outpatient treatment program in health posts of Arba Minch Zuria Woreda, Gamo zone, Southern Ethiopia: A retrospective cohort study
Source: PLoS One. 2020 Jun 30;15(6):e0234793. doi: 10.1371/journal.pone.0234793 (PMC7326160; doi:10.1371/journal.pone.0234793)
Supplement: S1 Data — (DOCX) [file pone.0234793.s001.docx]

| Data extraction tool: **Title of study: Predictors of time-to-recovery from severe acute malnutrition treated in an outpatient treatment program in health posts of Arba Minch Zuria woreda, Gamo zone, Southern Ethiopia: a retrospective cohort study**  Date of admission to OTP-----------dd/----------mm/-----------------yy  Date of discharge from OTP---------dd/----------mm/-----------------yy  To days Date -----------------dd/-------------------mm/---------------yy  Name of health post_______________________________Code No.___________  Guide to data collectors: Read the variables and fill the data abstraction form carefully by making appropriate circle or writing the response on the space provided accordingly. | | | | | | | | | | | | | | | | |
| --- | --- | --- | --- | --- | --- | --- | --- | --- | --- | --- | --- | --- | --- | --- | --- | --- |
| **S.no** | | **Part I: Patient characteristics** | | | | | | | | | **Skip to question** | | | | | |
| 101 | | Age at admission | | | ____________Months | | | | | |  | | | | | |
| 102 | | Sex | | | 1. Male 2. Female | | | | | |  | | | | | |
| 103 | | Distance to health post | | | 1. Less than 30 minutes 2. More than 30 minutes | | | | | |  | | | | | |
| 104 | | Breast feeding | | | 1. Yes 2. No | | | | | |  | | | | | |
| 105 | | Type of admission | | | 1. New 2. Admission after default | | | | | |  | | | | | |
| **Part II: Anthropometric characteristics** | | | | | | | | | | | | | | | | |
| 201 | | | Weight at admission | | (kg) | | | | | |  | | | | | |
| 202 | | | Weight at discharge | | (kg) | | | | | |  | | | | | |
| 203 | | | MUAC at admission | | (cm) | | | | | |  | | | | | |
| 204 | | | MUAC at discharge | | (cm) | | | | | |  | | | | | |
| **Part III: Co-morbidities during admission** | | | | | | | | | | | | | | | | |
| 301 | Admission criteria | | | | | 1. edema (Kwashiorkor) 2. Marasmus | | | | | |  | | | | |
| 302 | Does the child have co-morbidities during admission? | | | | | 1. Yes 2. No | | | | | |  | | | | |
| 303 | Does the child have fever during admission? | | | | | 1. Yes 2. No | | | | | |  | | | | |
| 304 | Does the child have Malaria during admission? | | | | | 1. Yes 2. No | | | | | |  | | | | |
| 305 | Does the child have cough during admission? | | | | | 1. Yes 2. No | | | | | |  | | | | |
| 306 | Does the child have diarrhea during admission? | | | | | 1. Yes 2. No | | | | | |  | | | | |
| 307 | If yes,, which type? | | | | | 1. Watery diarrhea 2. Bloody diarrhea | | | | | |  | | | | |
| 308 | Does the child have vomiting during admission? | | | | | 1. Yes 2. No | | | | | |  | | | | |
| 309 | Does the child have pneumonia during admission? | | | | | 1. Yes 2. No | | | | | |  | | | | |
| 310 | Does the child have anemia during admission? | | | | | 1. Yes 2. No | | | | | |  | | | | |
| **Part IV: Routine medication** | | | | | | | | | | | | | | | | |
| 401 | Was Vitamin A given? | | | | | 1. Yes 2. No 3. Not applicable | | | | | |  | | | | |
| 402 | Was Folic acid given? | | | | | 1. Yes 2. No | | | | | |  | | | | |
| 403 | Was deworming given? | | | | | 1. Yes 2. No 3. Not applicable | | | | | |  | | | | |
| 404 | Was Antibiotics given? | | | | | 1. Yes 2. No | | | | | |  | | | | |
| 405 | Was Measles vaccine given? | | | | | 1. Yes 2. No 3. Not applicable | | | | | |  | | | | |
| 406 | Was Anti-malarial given? (If malaria detected) | | | | | 1. Yes 2. No 3. Not applicable | | | | | |  | | | | |
| **Part V: Follow up** | | | | | | | | | | | | | | | | |
|  | Week | | | Admission (Week1) | | | 2 | | 3 | 4 | 5 | | 6 | | 7 | 8 |
| 501 | Weight(Kg) | | |  | | |  | |  |  |  | |  | |  |  |
| 502 | Weight change   1. Positive 2. Negative 3. Zero | | |  | | |  | |  |  |  | |  | |  |  |
| 503 | MUAC (cm) | | |  | | |  | |  |  |  | |  | |  |  |
| 504 | Oedema   1. Yes 2. No | | |  | | |  | |  |  |  | |  | |  |  |
| 505 | General danger sign   1. No 2. Yes | | |  | | |  | |  |  |  | |  | |  |  |
| 506 | Diarrihea   1. No 2. Yes | | |  | | |  | |  |  |  | |  | |  |  |
| 507 | Vomiting   1. No 2. Yes | | |  | | |  | |  |  |  | |  | |  |  |
| 508 | Fever   1. .No 2. Yes | | |  | | |  | |  |  |  | |  | |  |  |
| 509 | Cough   1. No 2. Yes | | |  | | |  | |  |  |  | |  | |  |  |
| 510 | Anemia(Palmar pallor)   1. No 2. Yes | | |  | | |  | |  |  |  | |  | |  |  |
| 511 | Skin infection   1. No 2. Yes | | |  | | |  | |  |  |  | |  | |  |  |
| 512 | Appetite test during follow up   1. Fail 2. Pass | | |  | | |  | |  |  |  | |  | |  |  |
| **Part VI: Outcome** | | | | | | | | | | | | | | | | |
| 601 | Recovery | | | | | | | 1. No 2. Yes | | | | | |  | | |
| 602 | Advert outcome | | | | | | | 1. Defaulter 2. Transferred 3. Non-respondent 4. Death 5. Unknown | | | | | |  | | |
| 603 | How much was the Mean weight gain? | | | | | | | _____________g/kg/day | | | | | |  | | |
| 604 | How much was the Average MUAC gain | | | | | | | ______________mm/day | | | | | |  | | |
| 605 | How many days did the patient stayed in the program? (Only for Recovered patients). | | | | | | | ____________Days | | | | | |  | | |

**END**

Name of data collector ____________________sign ________________date___________

Name of supervisor _____________________sign__________________date__________
